# Handheld SERS coupled with QuEChERs for the sensitive analysis of multiple pesticides in basmati rice

**DOI:** 10.1038/s41538-021-00117-z

**Published:** 2022-01-13

**Authors:** Natasha Logan, Simon A. Haughey, Lin Liu, D. Thorburn Burns, Brian Quinn, Cuong Cao, Christopher T. Elliott

**Affiliations:** 1 ASSET Technology Centre, Institute for Global Food Security, School of Biological Sciences, Queen’s University Belfast, 19 Chlorine Gardens, Belfast, BT9 5DL UK; 2 Material and Advanced Technologies for Healthcare, Queen’s University Belfast, 18-30 Malone Road, Belfast, BT9 5BN UK

**Keywords:** Nanoparticles, Characterization and analytical techniques

## Abstract

Pesticides are a safety issue globally and cause serious concerns for the environment, wildlife and human health. The handheld detection of four pesticide residues widely used in Basmati rice production using surface-enhanced Raman spectroscopy (SERS) is reported. Different SERS substrates were synthesised and their plasmonic and Raman scattering properties evaluated. Using this approach, detection limits for pesticide residues were achieved within the range of 5 ppb-75 ppb, in solvent. Various extraction techniques were assessed to recover pesticide residues from spiked Basmati rice. Quick, Easy, Cheap, Effective, Rugged and Safe (QuEChERs) acetate extraction was applied and characteristic spectral data for each pesticide was obtained from the spiked matrix and analysed using handheld-SERS. This approach allowed detection limits within the matrix conditions to be markedly improved, due to the rapid aggregation of nanogold caused by the extraction medium. Thus, detection limits for three out of four pesticides were detectable below the Maximum Residue Limits (MRLs) of 10 ppb in Basmati rice. Furthermore, the multiplexing performance of handheld-SERS was assessed in solvent and matrix conditions. This study highlights the great potential of handheld-SERS for the rapid on-site detection of pesticide residues in rice and other commodities.

## Introduction

Rice (*Oryza sativa*, “Queen of Grains”) is a staple food for more than 60% of the world’s population and its supply must more than double by 2050 to adequately feed growing populations^1,2^. Challenges for global rice production include agricultural water scarcity, urbanisation of farming land and climate change^3^. In addition, the COVID-19 pandemic has added a fourth dimension by disrupting the global food supply chain. Producers now face immense pressures from labour shortages and crop losses because of the pandemic. Rice is also continually under threat from pests (e.g. rice water weevil; *Lissorhoptrus oryzophilus*) which can consume between 5% and 20% of the crop^4^ and fungal diseases; rice blast (*Magnaporthe grisea*) and sheath blight (*Rhizoctonia solani*), which cause major production constraints in Asia^5^. Under favourable conditions fungal blight can cause up to a 50% decrease in rice crop yield annually around the world^6^.

Modern agricultural practices rely on synthetic chemicals, pesticides, fungicides and insecticides to improve rice yield by 20–50%^7^. Although valuable to food production, mixtures are commonly applied in excessive amounts, with less than 0.1% reaching intended targets^8^. Pesticide residues are persistent and pollute the environment (soil, air and groundwater) and contaminate the food chain, causing serious harm to wildlife and human health. Extremely toxic, carcinogenic and mutagenic residues can be adsorbed into the body through the skin, eyes, respiratory system and digestive system. Studies have shown a strong correlation between the accumulation of pesticide residues in the body and damage to the reproductive, nervous and immune systems, development of liver and cerebrovascular diseases, and cancers of the liver, gall bladder and breast^9^.

According to the Rapid Alert System for Food and Feed (RASFF)^10^ high levels of acephate (organophosphate), carbendazim (benzimidazole fungicide), thiamethoxam (neonicotinoid) and tricyclazole (fungicide) were frequently found above the Maximum Residue Limits (MRLs) in Basmati rice during the period between 2011 and 2020 (Supplementary Fig. 1a). A spike in notifications was also observed in 2014 for acephate and carbendazim residues in Basmati rice imported from India and Pakistan. Since 2018, notifications for thiamethoxam and tricyclazole in Basmati rice have been on the rise, suggesting the continuous development of novel agrochemicals (Supplementary Fig. 1b). Within the European Union (EU), MRLs for these residues in rice are all set at 0.01 mg/kg (ppm), which is significantly low compared to the regulatory limits set by other countries across the globe (Supplementary Table 1).

Conventional methods for pesticides in rice rely on liquid chromatography (LC) or gas chromatography (GC)-coupled with mass spectrometry (MS)^11^, which are highly accurate but can also require complex extractions, long analysis times and expensive instrumentation^12^. Therefore, it is difficult for these techniques to meet the requirements for on-site analysis thus, there is a clear need to develop rapid, cost-effective and portable techniques as screening tools. Surface-Enhanced Raman Spectroscopy (SERS) is a surface-sensitive technique in which inelastic light scattering of molecules is greatly enhanced (by up to 10^8^ or larger, enabling single-molecule detection in some cases), through the attachment or adsorption of target analytes close to, onto the surface or in between nano-roughened metallic surfaces or metallic nanoparticles (NPs) (so-called hot spots)^13^. SERS has been successfully employed previously for the detection of food and environmental contaminants such as, mycotoxins^14^, antibiotic residues^15^, mercury^16^ and tropane alkaloids^17^.

At present, developments have been made to successfully detect pesticide residues within various food matrices using SERS-based techniques. Chen *et al*. detected carbendazim residues in Oolong tea using gold nanoparticles (AuNPs) as substrates^18^. Liu et al. used a shell thickness-dependent Raman enhancement technique using silver-coated AuNPs (Au@Ag NPs), for the rapid detection of pesticides on various fruit peels^19^. Fu et al. developed a gold nanorod (GNR) array as a SERS substrate for the detection of thiabendazole in apple^20^. Li et al. used a SERS imaging technique to determine trace levels of thiophanate and its metabolite carbendazim in red bell pepper^21^. Wang et al. developed a gecko-inspired nanotentacle SERS (G-SERS) platform for the simultaneous detection of three pesticides in fruit and vegetables^22^. Whilst vital works they all focus on developing lab-based techniques with Raman spectrometer systems or microscopes. The on-site analysis of pesticide residues currently relies on enzymatic or colorimetric screening techniques which lack sensitivity, reliability and multiplexing capabilities. Consequently, producers rely on costly and lengthy confirmatory techniques, resulting in slow product turnaround.

Many extraction procedures have been previously developed for the analysis of pesticide residues in fruit and vegetables including swab techniques^23^ original Quick, Easy, Cheap, Effective, Rugged and Safe (QuEChERs) extraction^24^ and buffered QuEChERs^25^ (to help with the recovery of problematic acid- and base-sensitive pesticides). QuEChERs was originally designed for extraction and cleanup of pesticide residues from matrices with a high moisture content (>75%) and low-fat content^26^. Rice is considered a complex food matrix containing fatty acids, amino acids, dietary fibre, vitamins and other essential micronutrients^27^. Consequently, numerous non-targeted compounds are likely to be removed during solvent extractions such as, QuEChERs. Some works have successfully adapted the technique for grains and rice, however analysis mainly consists of LC-MS and GC-MS^28^, with very few focusing on SERS. Herein, several extraction procedures were examined to determine the most suitable method for pesticide recovery from Basmati rice when exploiting handheld-SERS for analysis.

Overall, this study presents vital improvements to the research area by developing a handheld-SERS-based platform to detect acephate, carbendazim, thiamethoxam and tricyclazole in Basmati rice using AuNP substrates. The novelty of the work lies in the combination of a handheld AuNP SERS-based technique with a QuEChERs extraction (more commonly applied to spectrometry techniques). As a result, this combination could facilitate SERS enhancement and improve sensitivities in matrix conditions. With future developments the technique could easily be adopted in-field thus providing a rapid, affordable and sensitive SERS-based platform. Overall, there is the potential to improve on-site applications for pesticide analysis in rice and with minor adaptions could also be applied to monitor other toxic contaminants (i.e., natural toxins), a range of food matrices (i.e., fruit and vegetables, cereals) or environmental samples (i.e., water and soil).

## Results

### Raman ‘fingerprint’ spectrum and molecular structure

**Fig. 1:**
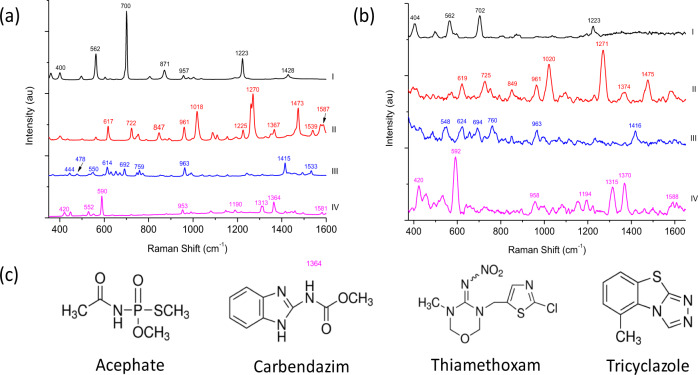
Raman scattering and molecular structures of pesticide residues. Raman ‘fingerprint’ spectra of (I) acephate (II) carbendazim (III) thiamethoxam and (IV) tricyclazole powder, obtained using (**a**) benchtop Raman microscope and (**b**) handheld Raman spectrometer (spectra is pre-treated using an advanced-averaging filter). (c) Molecular structures of compounds.

Raman spectral data was acquired on solid pesticide powder using a benchtop microscope and a handheld instrument, to characterise and ascertain their unique fingerprint spectra and main vibrational bands. Initially, both Raman techniques were used to determine the performance and accuracy of the handheld device, in comparison to the conventional Raman microscope. As expected, the spectral data from the Raman microscope clearly showed the main vibrational bands for each of the pesticides analysed (Fig. 1a). The results also confirmed that the handheld instrument could produce the same Raman (no SERS) characteristic peaks (Fig. 1b). Only minor shifts in the main vibrational bands were observed between the two techniques, i.e., from 700 to 702 cm^−1^, 1270 to 1271 cm^−1^, 1415 to 1416 cm^−1^ and 590 to 592 cm^−1^ for acephate (I), carbendazim (II), thiamethoxam (III) and tricyclazole (IV), respectively. In addition, some vibrational bands disappeared from the spectrum obtained using the handheld device however, slight changes are to be expected due to its reduced size and power output. A detailed description of each Raman band is highlighted in Supplementary Table 2. Overall, the results confirmed that the handheld device could successfully produce the Raman ‘fingerprint’ spectra for solid pesticide powder, with performance comparable to the conventional benchtop microscope.

### Synthesis and characterisation of colloidal nanogold substrates

**Fig. 2:**
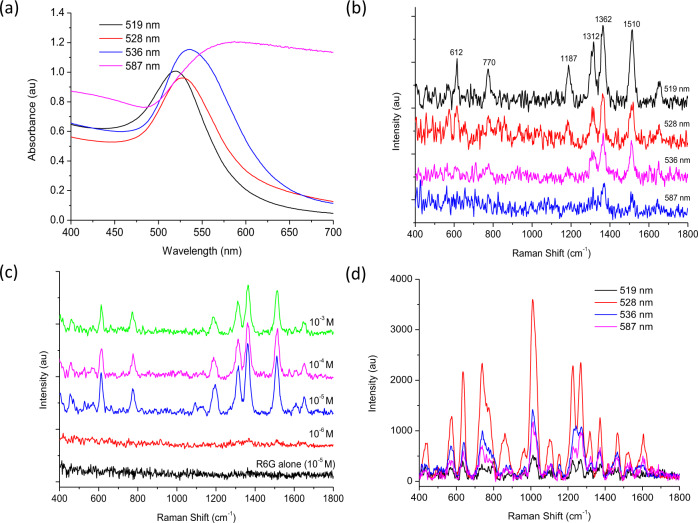
Characterisation of colloidal AuNP substrates and their corresponding SERS enhancement using handheld spectroscopy. **a** AuNP substrates with wavelength max. (λ_max_) 519 nm (black line), 528 nm (red line), 536 nm (blue line) and 587 nm (pink line). **b** SERS enhancement of AuNP substrates in the presence of Rhodamine 6 G (R6G) (10^−3 ^M). **c** SERS spectrum of AuNPs (λ_max_ = 519 nm) with decreasing concentrations of R6G (10^−3 ^M to 10^−6 ^M, green line to red line, respectively) and the Raman spectra of R6G alone (10^−5 ^M, black line) presenting no SERS. **d** AuNP substrates in the presence of carbendazim (100 ppm) and HCl (2 M).

AuNPs were synthesised using a well-established sodium-citrate reduction. Typically, particles with an average size of 10–20 nm and wavelength max. (λ_max_) at approx. 520 nm are produced using the Turkevich method^29^ whilst, the Frens method^30^ modifies the stoichiometric ratio between sodium citrate and Au, allowing a wider range of diameters to be produced (~10–100 nm). During synthesis the surface of colloidal AuNPs is passivated via chemisorption and/or physisorption of stabilising citrate ions^31^. The citrate ion shell remains loose on the surface of the particles and can be easily displaced by interactions such as gold–sulfur (Au–S) and gold–nitrogen (Au–N) bonding^32^. Due to the distance-dependence of electromagnetic field SERS enhancements (i.e., hot spots), substrates were synthesised using the Frens method to optimise the performance of handheld-SERS. Figure 2a illustrates broadening of SPR peak with the λ_max_ shifting from 519 nm (black line) to 587 nm (pink line), when the concentration of sodium citrate is decreased from 0.1% to 0.01%, respectively. Additionally, the results obtained through Dynamic Light Scattering (DLS) could confirm an increase in particle size from 25.4 nm to 81.8 nm by decreasing the concentration of sodium citrate (Supplementary Fig. 2), as has been observed by previous work^33^.

The SERS enhancement of the synthesised substrates was assessed by adsorbing a fluorescent dye; rhodamine 6G (RG6), onto the surface of the AuNPs prior to analysis. The main vibrational bands of RG6 were observed at 1312 cm^−1^, 1362 cm^−1^ and 1510 cm^−1^ (Fig. 2b) and are attributed to N–H bending, C–H bending and C–N stretching, respectively^34^. Full band assignments for RG6 can also be found in Supplementary Table 2. The results confirmed that the substrate with λ_max_ at 519 nm (Fig. 2b, black line) performed best as an enhancer with handheld-SERS. The Raman spectra of pure RG6 alone was not observed when the concentration of R6G was 10^−5 ^M (Fig. 2c, black line) however, in the presence of AuNPs (λ_max_ = 519 nm) the main vibrational bands could clearly be observed at the same concentration (Fig. 2c, blue line). At a higher concentration of R6G (10^−3 ^M) the SERS intensity slows, due to complete AuNP aggregation and saturation of ‘hot spots’ by the adsorbed dye (Fig. 2c, green line)^35^. Overall, the analytical enhancement factor (AEF) for Au substrate (λ_max_ = 519 nm) using handheld-SERS could be calculated as 8.04 × 10^3^, using the main characteristic peak of R6G at 1362 cm^−1^, in the absence and presence of AuNPs (Supplementary Eq. 1). Therefore, in this study, SERS scattering could be enhanced by 3 orders of magnitude, by utilising AuNPs and handheld-SERS.

Due to the distance-dependent properties of SERS, it was important to discover the enhancement which could be achieved in the presence of pesticides. Under suitable conditions, a reduction in inter-particle distance and the formation of ‘hot spots’ provides significant electromagnetic field enhancements thus allowing the unique ‘fingerprint’ spectrum for carbendazim to be distinguished using handheld-SERS (Fig. 2d). In the presence of carbendazim (100 ppm) and HCl (2 M), the synthesised AuNPs with λ_max_ at 528 nm displayed the greatest increase in SERS intensity (Fig. 2d, red line). However, with all other synthesised particles a reduction in SERS intensity was observed. AuNPs with λ_max_ at 519 nm displayed the greatest SERS enhancement with R6G (Fig. 2b, black line), but also exhibited the weakest SERS enhancement in the presence of carbendazim (Fig. 2d, black line). Thus, a larger SERS substrate was required to sufficiently reduce inter-particle distance and trap carbendazim molecules between adjacent nano-gaps. Overall, these results confirmed that AuNPs with λ_max_ at 528 nm were the most suitable SERS substrate for pesticide analysis and were used in all experiments hereafter.

### Optimisation of pesticide detection using handheld-SERS

**Fig. 3:**
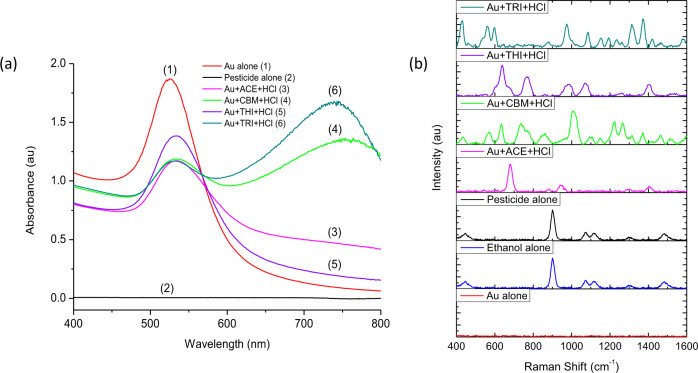
Stability of colloidal AuNPs (λ_max_ = 528 nm) and corresponding SERS spectra in the presence of pesticides and HCl (2 M). **a** UV–vis spectra of (1) Au alone, (2) pesticide alone, AuNPs in the presence of HCl (2 M) and (3) acephate (ACE), (4) carbendazim (CBM), (5) thiamethoxam (THI) and (6) tricyclazole (TRI). **b** SERS ‘fingerprint’ spectra for Au alone (red line), ethanol alone (blue line), pesticide alone (black line), AuNPs in the presence of HCl (2 M) and ACE (pink line), CBM (green line), THI (purple line) and TRI (dark cyan line). Pesticide alone (black line) is the Raman spectra for CBM at 100 ppm and confirms that no ‘fingerprint’ SERS spectra can be obtained unless AuNPs are present (green line).

Firstly, it was important to optimise all conditions for maximum SERS enhancement using the handheld device. A stock solution of carbendazim (CBM) at 100 ppm was prepared by dissolving in ethanol:dH_2_O (1:1, v/v). Experiment parameters were optimised including, Au substrate concentration, the ratio of pesticide to Au, the reagent (and volume) required for ‘hot spot’ formation and the required incubation time (Supplementary Fig. 3). Afterwards, UV–vis analysis was used to determine the stability of AuNPs in the absence and presence of pesticide residues (Fig. 3a). The results confirmed that without pesticides the AuNPs remained stable in solution, indicated by the distinct absorption peak at 528 nm (Fig. 3a, red line (1)). Colloidal AuNPs are stabilised by a coating layer of citrate ions on the surface, which are known to contain active organic functional groups (i.e., carboxylic and hydroxyl). As a result, the AuNPs exhibit a strong negative charge allowing the particles to remain stable in solution, electrostatically.

In the presence of pesticide residues and HCl (2 M), the inter-particle distance is reduced and the peak absorbance redshifts, resulting in a broadening of the absorption spectra (~650 nm). This can be explained by the displacement of citrate ions from the surface, shielding the repulsive forces between particles^36^. Furthermore, the addition of HCl lowers the pH of the surrounding medium, reducing the repulsive forces further. High concentrations of HCl can promote a greater degree of particle–particle interactions, leading to ‘fast’ aggregation. In this case, particles stick together during their first collision and form large aggregates rapidly, which can provide a strong increase in SERS intensity^37^. This degree of aggregation could be observed by the extreme broadening of bands after the addition of TRI (Fig. 3a, dark cyan line (6)) and CBM (Fig. 3a, green line (4)). However, to a lesser extent with the addition of ACE (Fig. 3a, pink line (3)) and THI (Fig. 3a, purple line (5)). Therefore, this result can also confirm that the interactions between pesticides and the Au surface also play a significant role in facilitating particle aggregation. However, predictions on SERS intensity cannot be made based on the degree of particle aggregation observed using UV–vis, as it is also possible to reach ‘aggregation saturation’ and reduced SERS intensity as a result. Therefore, it is fundamental to analyse this phenomenon using handheld-SERS.

SERS measurements were evaluated in the absence and presence of pesticide residues (Fig. 3b). The SERS results confirmed that AuNPs alone do not display a SERS spectrum (Fig. 3b, red line) and CBM alone only displays peaks coming from solvent. This is confirmed by the identical spectrum of pesticide alone and ethanol alone, shown in Fig. 3b (black line and blue line, respectively). However, when mixed with HCl (2 M) the typical ‘fingerprint’ SERS spectra for ACE (Fig. 3b, pink line), CBM (Fig. 3b, green line), THI (Fig. 3b, purple line) and TRI (Fig. 3b, dark cyan line) could be observed. Therefore, combining AuNPs (λ_max_ = 528 nm) and HCl (2 M) could provide sufficient electromagnetic field enhancements and ‘hot spot’ formation, allowing the detection of all four pesticide residues. The major SERS bands for all pesticides can also be observed clearly in the reference spectrum (Supplementary Fig. 4). Overall, the results confirmed that due to a combination of substrate selection, Au–pesticide interactions and the addition of HCl (2 M), pesticide residues could be detected and analysed using handheld-SERS.

### Detection of pesticide standards using handheld-SERS

**Fig. 4:**
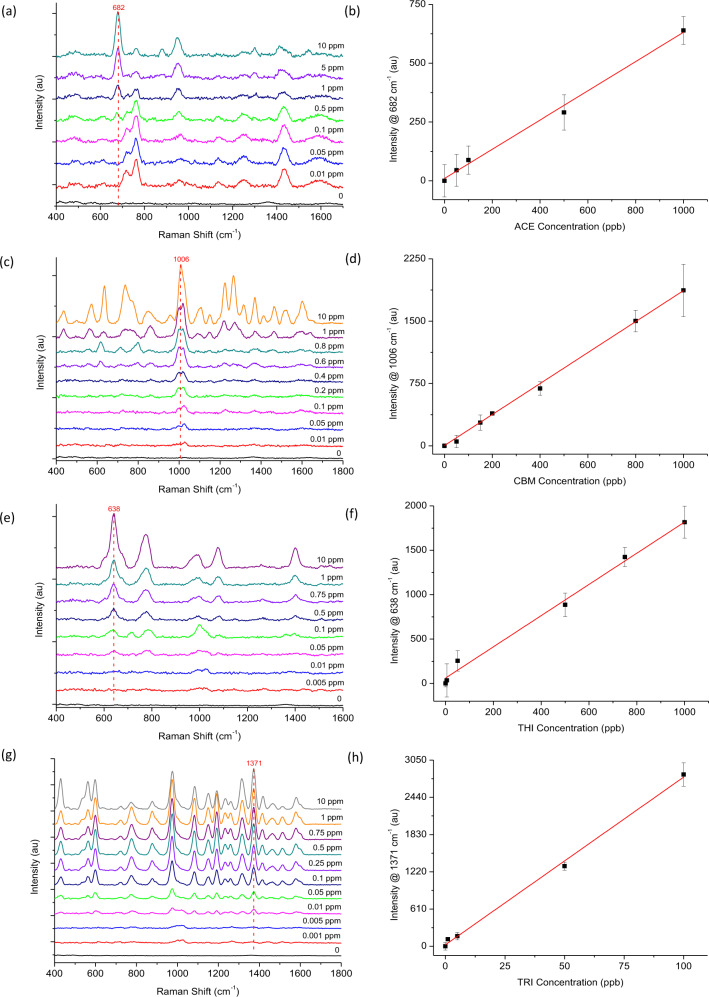
Analysis of pesticide standard solutions (in solvent) using handheld-SERS. **a**, **c**, **e**, **g** Spectrum of ACE, CBM, THI and TRI with increasing concentrations, respectively. **b**, **d**, **f**, **h** Linear relationship between increasing SERS intensity and the concentration of ACE, CBM, THI and TRI, respectively. All data was normalised relative to the blank (zero) and the standard deviation (σ) was calculated from triplicate samples (*n* *=* 21).

Pesticide standards were analysed using handheld-SERS to obtain the working range (Fig. 4). The results demonstrate enhanced SERS intensity in the presence of increasing pesticide concentrations for; ACE (Fig. 4a) CBM (Fig. 4c), THI (Fig. 4e) and TRI (Fig. 4g). Under optimum conditions the Raman instrument produced key spectral features at 682 cm^−1^, 1006 cm^−1^, 638 cm^−1^ and 1371 cm^−1^ for ACE, CBM, THI and TRI, which were visibly clear at concentrations 100 ppb, 50 ppb, 50 ppb and 10 ppb, respectively. These characteristic peaks allowed the relationship between peak intensity and pesticide concentration to be examined and quantified further. The limit of detection (LOD), limit of quantification (LOQ), linear fitting and R^2^ values for each pesticide are summarised in Supplementary Table 3. The LOD and LOQ are calculated using the IUPAC definition and formulas 3 S/M and 10 S/M, respectively, were S is the standard deviation of ten measurements of the blank solution, and M is the slope of the calibration curve^38^. The results confirmed a linear relationship between zero and 1 ppm for ACE (Fig. 4b), CBM (Fig. 4d) and THI (Fig. 4f) and between zero and 0.1 ppm for TRI (Fig. 4h) (Supplementary Table 3, *R*^2^ = 0.993-0.997). The LOD was calculated as 62 ppb, 47 ppb, 75 ppb and 5 ppb for ACE, CBM, THI and TRI, respectively. Therefore, these results confirmed that handheld-SERS could only detect below the recommended EU MRLs for TRI. One issue with the proposed mechanism is selectivity. As the chemical structure of pesticides differs greatly, interactions with the nanomaterial will also vary. For example, TRI is a sulfur containing organic molecule known to bind strongly to Au^39^, thus, it is not unexpected that the detection limits for this pesticide standard are lower. The results from handheld-SERS were validated against a benchtop Raman microscope (Supplementary Fig. 5). Linear fittings and detection limits were also obtained using the microscope (Supplementary Table 3, LOD = 0.3 ppb-5 ppb, *R*^2^ = 0.963–0.997) and compared to handheld-SERS. As expected, all pesticides were detected using the Raman microscope below the EU MRLs for Basmati rice, which are set at 10 ppb for ACE, CBM, THI and TRI by the European Commission^40^. Overall, the results confirmed the possibility to measure and quantify parts per billion (ppb) levels of pesticides using handheld-SERS. However, only TRI could be detected below the recommended MRLs thus, improvements to sensitivity are essential.

### Analysis of pesticide residues extracted from basmati rice

**Fig. 5:**
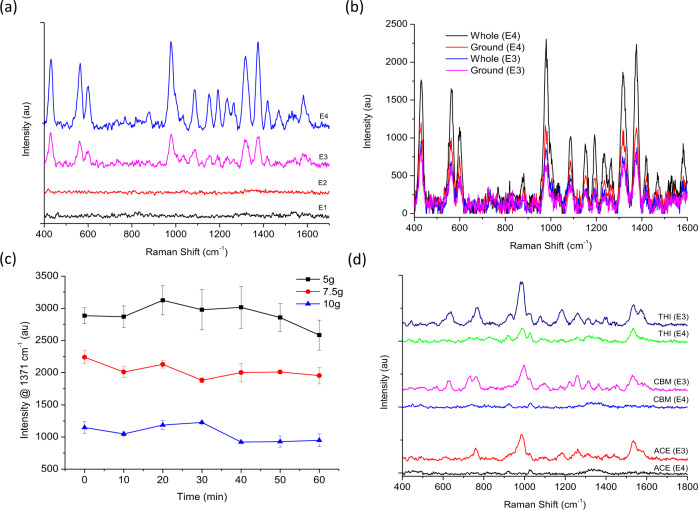
Optimisation of pesticide extraction from basmati rice. **a** Extraction of TRI from basmati rice using a swab technique (E1), solvent extraction (E2), QuEChERs acetate (E3) and original QuEChERs (E4). **b** Extraction of TRI from whole and ground basmati rice using QuEChERs acetate (E3) and original QuEChERs (E4). **c** Original QuEChERs extraction (E4) of TRI from basmati rice using different sample weights and a hydration step (*n* = 3). **d** Extraction of ACE, CBM and THI from basmati rice using QuEChERs acetate (E3) and original QuEChERs (E4). All analysis was conducted using handheld-SERS.

As TRI was most successfully detected during analysis of the standards, it was chosen to optimise the extraction procedure. Four extraction procedures were considered for the recovery of TRI intentionally spiked into basmati rice including, a swab extraction (E1), a solvent extraction (E2), QuEChERs acetate (E3) and original QuEChERs (E4) (Fig. 5a). The results confirmed that neither E1 (Fig. 5a, black line) or E2 (Fig. 5a, red line) could recover TRI from basmati rice, which may suggest that the residue has been absorbed by the matrix. However, both E3 (Fig. 5a, pink line) and E4 (Fig. 5a, blue line) could successfully extract TRI from basmati rice. To obtain the optimum conditions for the extraction both acetate (E3) and original (E4) QuEChERs were conducted using spiked whole and ground basmati rice (Fig. 5b). The results confirmed that TRI was recovered more efficiently from whole basmati rice using with the original QuEChERs (Fig. 5b, black line) which outperformed the QuEChERs acetate extraction (Fig. 5b, blue line). This may suggest that either rice is more absorbent in ground form; or more compounds are released from its complex composition when ground (i.e., fat or starch), thus enhancing matrix effects and reducing pesticide recovery. The low water content of uncooked rice (<14%) makes hydration crucial for extraction, to help facilitate interactions between the pesticide residue and extraction solvent^41^. Therefore, sample size and hydration time were evaluated using the original QuEChERs method (Fig. 5c). The results suggest that the recovery of TRI was most successful using a sample size of 5 g. Incubating the sample with dH_2_O did not improve the recovery, with recoveries remaining similar between zero and 20 min and declining afterwards. Therefore, incubation was not included as part of the final hydration step and the extraction solvent was added immediately after the addition of dH_2_O.

When the same optimised conditions were applied to recover ACE (Fig. 5d, black line), CBM (Fig. 5d, blue line) and THI (Fig. 5d, green line) from basmati rice, the extraction was not successful. However, when the original QuEChERs was replaced with QuEChERs acetate, these residues could be recovered and the unique ‘fingerprint’ spectra for ACE (Fig. 5d, red line), CBM (Fig. 5d, pink line) and THI (Fig. 5d, navy line) could once again be observed. “QuEChERS acetate” is considered the official method (2007.01) of the Association of Official Analytical Chemists for the determination of pesticide residues in food. The choice of using acidified acetonitrile allows the extraction of a wide range of pesticides with different polarities. The addition of salts, whilst reducing the volume of aqueous and promoting the ‘salting out’ effect, also together with acetic acid allows buffering of the extraction medium. Reducing the pH to ~5.0–5.5 allows the extraction of those pesticides which face stability problems or degradation^42^. The results can confirm that ACE, CBM and THI are unstable or degrade during the original QuEChERs extraction. Although these pesticides are more suited to an acidic medium the same was not true for TRI, which preferred the conditions of the original QuEChERs. As it was important to use an extraction suited to multiple pesticide residues, QuEChERs acetate could successfully extract all four pesticide residues from basmati rice and took less than 15 min to conduct. Therefore, QuEChERs acetate was selected for further analysis of pesticide recoveries using handheld-SERS.

**Fig. 6:**
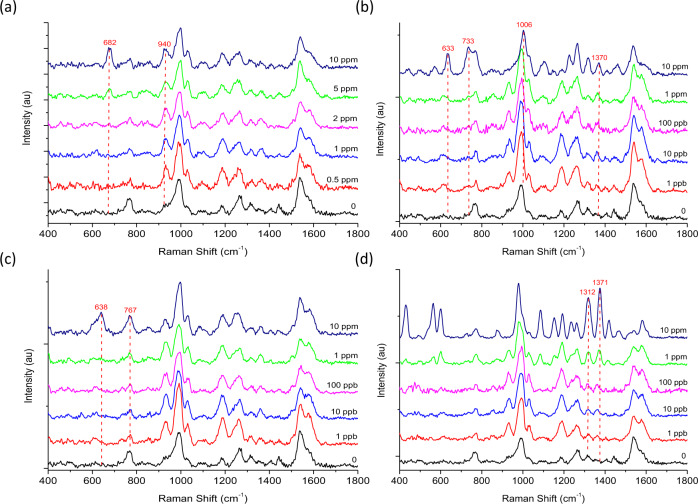
Pesticide residues at different concentrations detected in basmati rice using SERS. Spectrum of (**a**) ACE, (b) CBM, (**c**) THI and (**d**) TRI obtained using handheld-SERS after extracting from spiked basmati rice using QuEChERs acetate extraction.

Basmati rice was spiked with pesticide residues at concentrations ranging from 0.5 ppm to 10 ppm for ACE (Fig. 6a) and 1 ppb to 10 ppm for CBM (Fig. 6b), THI (Fig. 6c) and TRI (Fig. 6d). At lower concentrations, acephate could not be recovered as easily and was therefore spiked at higher concentrations to find the lowest detectable limit. The results confirmed that the characteristic SERS vibrational bands for ACE, CBM, THI and TRI could be observed at 682 cm^−1^, 1006 cm^−1^, 638 cm^−1^ and 1371 cm^−1^ respectively, matching those positions observed when analysing the standards (Fig. 4). However, noticeable peaks at 760 cm^−1^, 990 cm^−1^, 1180 cm^−1^, 1260 cm^−1^ and 1535 cm^−1^ were also confirmed in the unspiked rice sample, which may be attributed to the extraction of non-targeted compounds and matrix effects from the extraction medium. Therefore, for quantification, any characteristic peaks within range of the background signal were avoided. CBM was the only pesticide examined with spectral features in close proximity of the background. The peak at 633 cm^−1^ for CBM was chosen for analysis, as the original peak at 1006 cm^−1^ was considered too close to that of 990 cm^−1^. Therefore, based on the characteristic peaks at 682 cm^−1^, 633 cm^−1^, 638 cm^−1^ and 1371 cm^−1^ the lowest concentration visually detectable was 1 ppm, 1 ppb, 1 ppb and 1 ppb for ACE, CBM, THI and TRI, respectively. There was also a linear relationship between the concentration of pesticides extracted from basmati rice and increasing SERS intensity (Supplementary Fig. 6 and Supplementary Table 3, LOD = 0.6 ppb-800 ppb, *R*^2^ = 0.918–0.988). Additionally, the detection limits were compared to those obtained using the benchtop Raman microscope (Supplementary Fig. 7) and the results were almost identical between the two instruments (Supplementary Table 3, LOD = 0.3 ppb-800 ppb, *R*^2^ = 0.937–0.996).

The results also indicated that the QuEChERs acetate extraction could help to improve sensitivity in matrix conditions, from those obtained during analysis of the standards. As a result, CBM, THI and TRI could be detected below the EU MRL of 10 ppb in basmati rice; however, the sensitivity for ACE could not be improved beyond 800 ppb. The improvement to sensitivity in matrix conditions was attributed to the high ionic strength (44%, w/v) and low pH (5.0) of the extraction medium. As reported previously, bare-AuNPs lack stability in high electrolyte environments (>0.35%)^43^ and acidic conditions (<pH 7.4)^44^. In these conditions, citrate is protonated thus, the negative charges on the particle surface are greatly reduced, resulting in aggregation and increased clustering. Therefore, the conditions of the extraction are favourable for accelerating aggregation and ‘hot spot’ formation. As a result, lower concentrations of pesticide can become trapped within adjacent nano-gaps and are therefore detectable using handheld-SERS. In the case of ACE, it is often difficult to extract as it degrades easily and requires different conditions compared to other compounds such as, the use of low temperatures, problems which have also become apparent during GC-MS analysis of the compound^42^. To confirm that the analytical performance of our method is comparable to, or an improvement on previous techniques reported in the literature, several parameters have been compared for the detection of ACE, CBM, THI and TRI in rice (Supplementary Table 4). Whilst sensitive and rapid methods have been reported, the novelty of this work lies in its unique sensing mechanism thus, helping to improve portability and multiplexing capabilities in matrix conditions.

To evaluate the repeatability and reliability of the QuEChERs acetate extraction the % recovery and RSD (%) were obtained by spiking basmati rice with individual pesticides at three different concentrations. The average recovery and RSD of the extractions were within the range 83.4% to 115.0% and 3.6% to 23.8%, respectively (Table 1). Furthermore, the RE_accuracy_ (%) was calculated to be in the range of −17% to 4.5%, therefore, the results could confirm that some of the residues were not completely released during the extraction (Supplementary Table 5). Additionally, the results were compared by analysing the extracted residues with the Raman microscope and similar results were observed (Supplementary Table 5, Recovery = 83.4% to 118.7%, RSD = 3.0% to 22.9%, RE _accuracy_ = −16.6% to 19%). Finally, the RE_precision_ can be used to directly compare the performance of the two techniques; handheld-SERS and the Raman microscope. As the results were all close to zero (−0.14 to 0.33, Supplementary Table 5) this can confirm that there was no significant difference between the two techniques for analysing pesticide residues extracted from basmati rice using QuEChERs acetate. Therefore, using this approach the performance of handheld-SERS could be significantly improved. Overall, the results confirmed that pesticide residues could be successfully extracted and detected from basmati rice. Using a combination of QuEChERs acetate extraction and handheld-SERS allows rapid and straightforward detection. Therefore, with improvements there is the potential for handheld-SERS to replace other methods of in-field testing or to be used as a tier one screening tests (as has been discussed previously in the fight against food fraud^45,46^), ahead of confirmatory laboratory techniques such as, LC-MS or GC-MS. Developments to these methodologies will help to improve food safety by enhancing the on-site analysis of toxic contaminants within key food groups.

**Table 1:** Recovery and validation of extracted pesticide residues from basmati rice using QuEChERs acetate.

Pesticide	Spiked concentration (mg/kg, ppm)	Extracted concentration (mg/kg, ppm)	Recovery (%)	RSD (%)
ACE	1	1.02	102.4	14.7
	2	2.30	115.0	7.4
	10	10.48	104.8	3.6
CBM	0.001	0.0008	83.4	11.1
	0.1	0.090	90.0	20.5
	10	9.45	94.5	9.6
THI	0.001	0.0009	91.1	23.8
	0.1	0.097	96.5	11.1
	10	9.81	98.1	4.1
TRI	0.001	0.0011	104.5	12.3
	0.1	0.0997	99.7	15.0
	10	9.91	99.1	8.9

### Multiplex analysis of pesticide residues in solvent and basmati rice

**Fig. 7:**
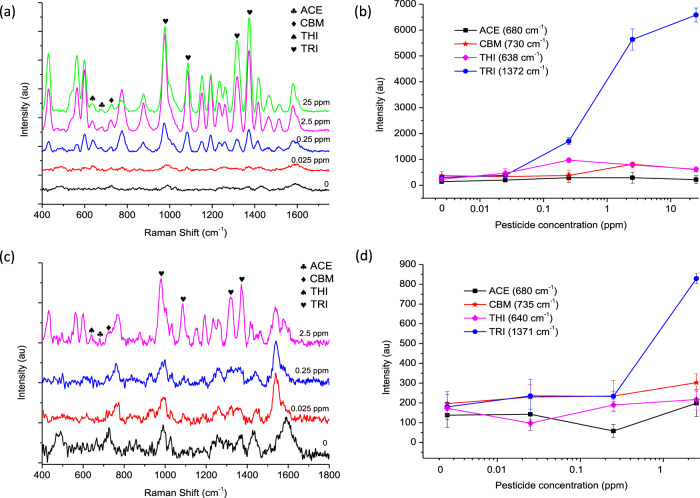
Analysis of mixed pesticide residues (ACE, CBM, THI and TRI at equal concentrations) using handheld-SERS. **a** SERS spectrum for mixed pesticide residues analysed in solvent. **b** Identified spectral features and corresponding SERS intensity for individual pesticide residues in solvent. **c** SERS spectrum for mixed pesticide residues extracted from spiked basmati rice. **d** Identified spectral features and corresponding SERS intensity for individual pesticide residues extracted from spiked basmati rice.

In agricultural practices, pesticide exposure is widespread and vast amounts are applied as chemical mixtures. Additionally, some pesticides (e.g., aldrin, dichlorodiphenyltrichloroethane (DDT) and hexachlorobenzene) contain persistent organic pollutants (POPs) which can resist degradation thus, remain in the environment for many years and have the ability to bioaccumulate and biomagnify within the food chain^9^. Therefore, more efforts are required to improve the multiplex analysis of pesticide residues. To identify if this SERS approach was applicable, the simultaneous detection of ACE, CBM, THI and TRI was analysed as a standard mixture (Fig. 7a, b) and the mixture was subsequently spiked and extracted from basmati rice using QuEChERs acetate (Fig. 7c, d). All four pesticides were mixed at equal concentrations thus, the concentrations reported are the concentrations recovered for each of the four pesticides. The characteristic peaks for ACE (680 cm^−1^), CBM (730 cm^−1^ and 735 cm^−1^), THI (638 cm^−1^ and 640 cm^−1^) and TRI (1318 cm^−1^ and 1372 cm^−1^) could all be visually identified at concentrations 0.25 ppm and 2.5 ppm as a standard mixture (Fig. 7a, blue line) and from spiked basmati rice (Fig. 7c, pink line), respectively. However, in both cases many of the strong vibrational bands observed within the spectral region of 1000–1600 cm^−1^ were attributed to TRI (Fig. 7a, c, hearts). Due to the strong signal response of TRI, only small peaks were therefore decipherable for ACE (Fig. 7a and c, clubs), CBM (Fig. 7a, c, diamonds) and THI (Fig. 7a, c, spades), respectively. Analysis of the spectral features for each pesticide individually highlights the suppression of characteristic peaks for ACE, CBM and THI in both the standard (Fig. 7b) and spiked basmati rice (Fig. 7d) conditions. TRI has higher affinity to the Au substrate (due to Au–S bonds) thus, a strong relationship between increasing SERS intensity and TRI concentration can be observed in both standard conditions (Fig. 7b, blue line) and spiked basmati rice (Fig. 7d, blue line).

The results highlight that the detection of TRI in a complex rice sample using this approach can be achieved however, the presence of other pesticides may be difficult to quantify. SERS signals for analytes within a complex matrix can be suppressed by other analytes which are highly Raman active, have higher binding affinity to the substrate or which saturate SERS ‘hot spot’ positions. A known problematic area for SERS is spectral overlapping which will need crucial attention if multiplex analyte analysis is to improve. Areas for improvement may include: 1) enhancing the specificity of the Au substrate, which will ultimately help to reduce matrix effects, the detection of non-targeted compounds and interactions between those analytes which cannot bind as strongly to the substrate; 2) reduce spectral overlapping and interference between multiple residues in a sample, through the use of artificial intelligence (AI) and/or machine learning algorithms e.g. self-modelling mixture analysis (SMA)^47^ or convolutional neural networks (CNN)^48^. These will be important steps forward to improve the rapid on-site analysis of multiple contaminants within food and environmental samples.

## Discussion

A rapid, handheld technique for the extraction of four pesticide residues from basmati rice has been developed. Selection of suitable AuNP substrates allowed maximum handheld-SERS enhancement. Sensitivities were improved by incorporation of a modified QuEChERs extraction, not commonly employed for SERS. The approach could detect concentrations extracted from spiked basmati rice within the range 0.6–800 ppb. Three out of four pesticides in matrix conditions could be detected below the MRL of 10 ppb in rice, set by the EU Commission. The average recoveries and RSD values were calculated within the range of 83.4% to 115.0% and 3.6% to 23.8%, respectively. Additionally, the results were validated against a benchtop Raman microscope. Multiplex analysis using handheld-SERS allowed pesticide concentrations in solvent and basmati rice to be detected at 0.25 ppm and 2.5 ppm, respectively. Overall, the results confirmed that there is potential for AuNP substrates, combined with QuEChERs extraction and handheld-SERS to successfully detect pesticide residues in basmati rice. Future developments to substrate design and the incorporation of machine learning algorithms may help to improve sensitivities, spectral overlapping and multiplexing performance in the future. Thus, there is substantial merit in the approach as a tier one screening tool, for the on-site analysis of pesticide residues in rice, agricultural crops (i.e., grains, fruit, vegetables) and environmental samples (i.e. groundwater and soil).

## Methods

### Analysis instrumentation

Ultraviolet-visible spectroscopy (UV–vis) measurements were performed using a Cary 60 spectrophotometer (Agilent Technologies, USA). Handheld-SERS measurements were performed using a HRS-30 spectrometer equipped with a 785 nm laser and Raman fibre optic probe operated at 400 mW (80% laser power) at an integration time of 5 s (Ocean Insight, USA). Benchtop SERS measurements were also carried out using a DXR2 Raman Microscope (ThermoFisher Scientific, UK) operated with an excitation laser light at 785 nm, laser power of 24 mW, integration time of 5 s, ×10 objective lens within the spectral range of 400–1600 cm^−1^. Spectral data was averaged (*n* = *3*), smoothed using Savitzky-Golay filtering and fitted with OriginPro 8.5 software. AuNP size measurements were conducted using a Zetasizer NanoZS (Malvern, UK).

### Chemicals and reagents

Sodium citrate tribasic dihydrate (HOC(COONa) (CH_2_COONa)_2_·aq), gold (III) chloride trihydrate (HAuCl_4_·3H_2_O_,_ 99.9%), acephate (O,S-Dimethyl N-acetylphosphoramidothioate), carbendazim (Methyl benzimidazol-2-ylcarbamate), thiamethoxam (3-(2-Chloro-5-thiazolylmethyl)tetrahydro-5-methyl-*N*-nitro-4*H*-1,3,5-oxadiazin-4-imin), tricyclazole (8-methyl-[1,2,4]triazolo[3,4-b][1,3]benzothiazole), rhodamine 6G (R6G), hydrochloric acid 37% (HCl), acetic acid ≥99% (HAc), acetonitrile ≥99.9% (MeCN), absolute ethanol ≥99.8%, magnesium sulphate (MgSO_4_), sodium chloride (NaCl), sodium acetate (NaOAc), potassium chloride (KCl), 96-well flat bottom ELISA plates and polyester foam tipped sterile swabs were all purchased from Sigma Aldrich (UK). Indian basmati rice samples were produced and supplied by Green Saffron (Cork, Ireland).

### Synthesis of colloidal nanogold substrates

Spherical nanogold was synthesised using a previous method^29^ with minor adjustments for regulation of particle size^30^. Briefly, 1 mM HAuCl_4_ was dissolved in 99 mL of deionised water (dH_2_O) and heated until rapidly boiling. Upon reflux 10 mL, 5 mL, 2.5 mL or 1.75 mL of 1% sodium citrate in dH_2_O was quickly added to the boiling solution, under vigorous stirring. The solution was removed from the heat after the colour changed from yellow to wine-red/purple-red/purple-brown/murky-brown which indicates the citrate reduction of gold ions, followed by DLS analysis to confirm particle size.

### Analysis of pesticide standard solutions using handheld-SERS

Stock solutions of pesticide residues were first prepared by dissolving in a relative solvent (ethanol or dH_2_O) and stored at 4 °C. All further dilutions of the stock solution were prepared from 0.001 ppm to 10 ppm in dH_2_O. In a typical experiment, minor adjustments were made to a previous method^49^ and optimised conditions for analysis were as follows; 1 mL of AuNPs (528 nm, OD_528 nm_ = 3.0) was added to a clear glass vial followed by 50 µL of pesticide at varying concentrations. Finally, 5 µL of HCl (2 M) was added to the mixture, inverted several times and incubated at room temperature for 2 min. For comparison, the same experimental conditions were applied to a 96-well ELISA plate for analysis with a Raman microscope.

### Extraction and analysis of pesticide residues in basmati rice using handheld-SERS

Prior to spiking, basmati rice samples were cleaned thoroughly by rinsing in tap water, dH_2_O and air dried. For extraction 1 (E1) and extraction 2 (E2) basmati rice samples were spiked by weighing 5 g of dried basmati rice into a clean plastic weigh boat and mixing with 1 mL of pesticide solution at concentrations ranging from 1-100 ppm. After mixing several times with a spatula over the course of 30 min, the samples were left to completely dry at room temperature overnight (~12 h). For extraction 3 (E3) and extraction 4 (E4), 5 g of dried basmati rice was weighed into a 50 mL centrifuge tube and spiked with 2 mL of pesticide solution at concentrations ranging from 10 ppb-100 ppm. The samples were placed on a roller for 30 min and left to completely dry at room temperature overnight (~12 h). The four extraction procedures were conducted as follows:

Swab extraction (E1): Swab sticks pre-soaked in ethanol (EtOH) were drawn through the rice samples evenly for ~90 s and immersed in 1 mL of the extraction solvent (EtOH). The swab tip was removed and vortexed in EtOH for ~30 s to release pesticide residues. Subsequently, 50 µL of the extracted residues were removed for SERS analysis.

Solvent extraction (E2): 1 g of spiked basmati rice was placed directly into a 2 mL plastic Eppendorf tube with 1 mL of EtOH. The sample was vortexed for ~30 s to release pesticide residues and 50 µL was removed for SERS analysis.

QuEChERs acetate (E3) and original QuEChERs (E4): QuEChERs extractions were conducted with minor adaptions to a previous report^50^. Due to the low water content of rice, water was first added (1:1, 5 mL) to hydrate the sample, followed by 15 mL of extraction solvent (MeCN, with the addition of 1% HAc for E3) for single-phase extraction of the sample and vortexed for 1 min. Liquid-liquid partitioning was performed with the addition of salts; 7 g of MgSO_4_ and 1.8 g NaOAc (E3) or 4 g of MgSO_4_ and 1 g of NaCl (E4) and vortexed for 1 min followed by a 5 min centrifugation at 1970 x*g* (RCF). Subsequently, 10 mL of the supernatant was removed and analysed using the handheld spectrometer and benchtop microscope.

For all extractions, SERS analysis was conducted using the method described in the section ‘analysis of pesticide standard solutions using handheld-SERS’. Due to the hazardous nature of the chemicals used during the procedures, all waste was collected and disposed of accordingly.

### Determination of extraction recovery, release factor and matrix effects

For QuEChERs, initially the concentration of pesticide extracted was calculated using Eq. (1);

1 $$C_{{{{{\mathrm{R}}}}}}\left( {{{{{{\mathrm{ppb}}}}}}} \right) = \frac{{C_{{{{{{\mathrm{SS}}}}}}}\left( {{{{{{\mathrm{ppb}}}}}}} \right) \times V_{{{{{{\mathrm{SS}}}}}}}\left( {{{{{{\mathrm{mL}}}}}}} \right)}}{{V_{{{{{{\mathrm{ES}}}}}}}\left( {{{{{{\mathrm{mL}}}}}}} \right)}}$$

were the *C*_R_ is the concentration of pesticide recovered, *C*_SS_ is the concentration of spiking solution, *V*_SS_ is the volume of spiking solution (mL) and *V*_ES_ is the volume of extraction solvent including dH_2_O (mL) (ignoring the small amount of water that will be absorbed by the matrix). Using Eq. (1) the concentration of pesticide extracted using this approach will be diluted 1:10 from the original spiking concentration. Secondly, it cannot be assumed that 100% of the spiked concentration will be extracted during the process. To determine the amount recovered and assess matrix effects, the release factor (RF) (%) was calculated using Eq. (2);

2 $${{{\rm{RF}}}}\;({{{{{\mathrm{\% }}}}}}) = \frac{{M_{{{{\rm{SERS}}}}}\left( {{{{{{\mathrm{ppm}}}}}}} \right) - m_{{{{\rm{SERS}}}}}({{{{{\mathrm{blank}}}}}})}}{{S_{{{{\rm{SERS}}}}}\left( {{{{{{\mathrm{ppm}}}}}}} \right) - s_{{{{\rm{SERS}}}}}({{{{{\mathrm{blank}}}}}})}}$$

were the *M*_SERS_ is the SERS intensity at a certain concentration spiked in matrix, *m*_SERS_ is the SERS intensity of the unspiked matrix, *S*_SERS_ is the SERS intensity of the same concentration spiked in solvent and s_SERS_ is the SERS intensity of the unspiked solvent. The RF (%) was calculated for each pesticide using a prominent SERS peak typical to each pesticide and was between the range 55% and 79%. Therefore, from our calculations 21–45% was either not released in the extraction or not detected using handheld-SERS due to matrix interferences.

### Reporting summary

Further information on research design is available in the Nature Research Reporting Summary linked to this article.

## Supplementary information

Supplementary Information (Clean)

REPORTING SUMMARY

## Data Availability

The datasets generated during the study are available from the corresponding author upon reasonable request.

## References

[1] Yadav SC, Mukerjee SC, Netam RS (2018). Efficacy of tricyclazole with integrated disease management against the disease control and yield improvement of rice. Int. J. Chem. Stud..

[2] Food and Agriculture Organization of the United Nations (FAO). How to feed the World in 2050. 2009. Available at: http://www.fao.org/fileadmin/templates/wsfs/docs/expert_paper/How_to_Feed_the_World_in_2050.pdf (last accessed June 2021).

[3] Asibi EA, Chai Q, Coulter AJ (2019). Rice blast: a disease with implications for global food security. Agron.

[4] Deutsch CA (2018). Increase in crop losses to insect pests in a warming climate. Science.

[5] Gnanamanickam, S. S. In *Biological Control of Rice Diseases* Vol. 8, Ch. 2 (eds. Cônsoli, F. L. Parra, J. R. P. & Zucchi, R. A.) 13–42 (Springer, 2009).

[6] Zheng A (2013). The evolution and pathogenic mechanisms of the rice sheath blight pathogen. Nat. Commun..

[7] Huang S, Hu J, Guo P, Liu M, Wu R (2015). Rapid detection of chlorpyriphos residue in rice by surface-enhanced Raman scattering. Anal. Methods.

[8] Xu M-L, Gao Y, Han XX, Zhao B (2017). Detection of pesticide residues in food using surface-enhanced Raman spectroscopy: a review. J. Agric. Food Chem..

[9] Kim K-H, Kabir E, Jahan SA (2017). Exposure to pesticides and the associated human health effects. Sci. Total Environ..

[10] European Commission, Rapid Alert System for Food and Feed (RASFF) Portal Available at: https://webgate.ec.europa.eu/rasff-window/portal/ (last accessed June 2021).

[11] Pareja L, Fernández-Alba AR, Cesio V, Heinzen H (2011). Analytical methods for pesticide residues in rice. Trends Anal. Chem..

[12] Hernández F, Sancho JV, Pozo OJ (2005). Critical review of the application of liquid chromatography/mass spectrometry to the determination of pesticide residues in biological samples. Anal. Bioanal. Chem..

[13] Langer J (2021). Present and future of surface-enhanced Raman scattering. ACS Nano.

[14] Li JJ, Yan H, Tan XC, Lu ZC, Han HY (2019). Cauliflower-inspired 3D SERS substrate for multiple mycotoxins detection. Anal. Chem..

[15] Dhakal S (2018). A simple surface-enhanced Raman spectroscopic method for on-site screening of tetracycline residue in whole milk. Sensors.

[16] Logan N, Lou-Franco J, Elliott C, Cao C (2021). Catalytic gold nanostars for SERS-based detection of mercury ions (Hg^2+^) with inverse sensitivity. Environ. Sci.: Nano.

[17] Lu J (2019). Silver nanoparticle-based surface-enhanced Raman spectroscopy for the rapid and selective detection of trace tropane alkaloids in food. ACS Appl. Nano Mater..

[18] Chen X (2019). Detection and quantification of carbendazim in Oolong tea by surface-enhanced Raman spectroscopy and gold nanoparticle substrates. Food Chem..

[19] Liu B (2012). Shell thickness-dependent Raman enhancement for rapid identification and detection of pesticide residues at fruit peels. Anal. Chem..

[20] Fu G, Sun D-W, Pu H, Wei Q (2019). Fabrication of gold nanorods for SERS detection of thiabendazole in apple. Talanta.

[21] Li JL, Sun DW, Pu H, Jayas DS (2017). Determination of trace thiophanate-methyl and its metabolite carbendazim with teratogenic risk in red bell pepper (Capsicumannuum L.) by surface-enhanced Raman imaging technique. Food Chem..

[22] Wang P (2017). Gecko-inspired nanotentacle surface-enhanced Raman spectroscopy substrate for sampling and reliable detection of pesticide residues in fruits and vegetables. Anal. Chem..

[23] He L, Chen T, Labuza TP (2014). Recovery and quantitative detection of thiabendazole on apples using a surface swab capture method followed by surface-enhanced Raman spectroscopy. Food Chem..

[24] Anastassiades M, Lehotay S, štajnbaher D, Schenck F (2003). Fast and easy multiresidue method employing acetonitrile extraction/partitioning and “dispersive solid-phase extraction” for the determination of pesticide residues in produce. J. AOAC Int..

[25] Lehotay SJ, Mastovská K, Lightfield AR (2005). Use of buffering and other means to improve results of problematic pesticides in a fast and easy method for residue analysis of fruits and vegetables. J. AOAC Int..

[26] Lehotay SJ (2010). Comparison of QuEChERS sample preparation methods for the analysis of pesticide residues in fruits and vegetables. J. Chromatogr. A.

[27] Liu Z (2017). Multi-pesticides residue analysis of grains using modified magnetic nanoparticle adsorbent for facile and efficient cleanup. Food Chem..

[28] Hou X, Han M, Dai X, Yang X, Yi S (2013). A multi-residue method for the determination of 124 pesticides in rice by modified QuEChERS extraction and gas chromatography–tandem mass spectrometry. Food Chem..

[29] Turkevich J, Stevenson PC, Hillier J (1951). A study of the nucleation and growth processes in the synthesis of colloidal gold. Discuss. Faraday Soc..

[30] Frens G (1973). Controlled nucleation for the regulation of the particle size in monodisperse gold suspensions. Nat. Phys. Sci..

[31] Chansuvarn W, Tuntulani T, Imyim A (2015). Colorimetric detection of mercury(II) based on gold nanoparticles, fluorescent gold nanoclusters and other gold-based nanomaterials. Trends Anal. Chem..

[32] Zhang Y, McKelvie ID, Cattrall RW, Kolev SD (2016). Colorimetric detection based on localised surface plasmon resonance of gold nanoparticles: Merits, inherent shortcomings and future prospects. Talanta.

[33] Luo H, Huang Y, Lai K, Rasco BA, Fan Y (2016). Surface-enhanced Raman spectroscopy coupled with gold nanoparticles for rapid detection of phosmet and thiabendazole residues in apples. Food Control.

[34] Jensen L, Schatz GC (2006). Resonance Raman scattering of rhodamine 6G as calculated using time-dependent density functional theory. J. Phys. Chem. A.

[35] Gorbachevskii MV (2018). Amplification of surface-enhanced Raman scattering by the oxidation of capping agents on gold nanoparticles. RSC Adv..

[36] Rana K, Bhamore JR, Rohit JV, Park T-J, Kailasa SK (2018). Ligand exchange reactions on citrate-gold nanoparticles for a parallel colorimetric assay of six pesticides. N. J. Chem..

[37] Akanny E (2019). Development of uncoated near-spherical gold nanoparticles for the label-free quantification of Lactobacillus rhamnosus GG by surface-enhanced Raman spectroscopy. Anal. Bioanal. Chem..

[38] Long G, Winefordner JD (2008). Limit of detection. A closer look at the IUPAC definition. Anal. Chem..

[39] Häkkinen H (2012). The gold–sulfur interface at the nanoscale. Nat. Chem..

[40] European Commission pesticide database, Available at https://ec.europa.eu/food/plant/pesticides/eu (last accessed June 2021).

[41] Barci PEP (2020). Modified QuEChERS method for multiresidue determination of pesticides in pecan nuts by liquid chromatography tandem mass spectrometry. Food Anal. Methods.

[42] Rizzetti TM (2016). Optimization of a QuEChERS based method by means of central composite design for pesticide multiresidue determination in orange juice by UHPLC–MS/MS. Food Chem..

[43] Logan N, McVey C, Elliott C, Cao C (2020). Amalgamated gold-nanoalloys with enhanced catalytic activity for the detection of mercury ions (Hg^2+^) in seawater samples. Nano Res..

[44] Park S (2019). Reversibly pH-responsive gold nanoparticles and their applications for photothermal cancer therapy. Sci. Rep..

[45] Black C, Haughey SA, Chevallier OP, Galvin-King P, Elliott CT (2016). A comprehensive strategy to detect the fraudulent adulteration of herbs: The oregano approach. Food Chem..

[46] Wielogorska E (2018). Development of a comprehensive analytical platform for the detection and quantitation of food fraud using a biomarker approach. The oregano adulteration case study. Food Chem..

[47] Hu B, Sun D-W, Pu H, Wei Q (2020). Rapid nondestructive detection of mixed pesticides residues on fruit surface using SERS combined with self-modeling mixture analysis method. Talanta.

[48] Zhu J (2021). Rapid on-site identification of pesticide residues in tea by one-dimensional convolutional neural network coupled with surface-enhanced Raman scattering. Spectrochim. Acta A: Mol. Biomol. Spectrosc..

[49] Dowgiallo AM, Guenther DA (2019). Determination of the limit of detection of multiple pesticides utilizing gold nanoparticles and surface-enhanced Raman spectroscopy. J. Agric. Food Chem..

[50] Pareja L, Cesio V, Heinzen H, Fernández-Alba AR (2011). Evaluation of various QuEChERS based methods for the analysis of herbicides and other commonly used pesticides in polished rice by LC–MS/MS. Talanta.

