# Supplementary material for: Handheld SERS coupled with QuEChERs for the sensitive analysis of multiple pesticides in basmati rice
Source: NPJ Sci Food. 2022 Jan 13;6:3. doi: 10.1038/s41538-021-00117-z (PMC8758682; doi:10.1038/s41538-021-00117-z)
Supplement: Supplementary file 1 — Supplementary Information (Clean) [file 41538_2021_117_MOESM1_ESM.docx]

**Supplementary Information**

Handheld SERS Coupled with QuEChERs for the Sensitive Analysis of Multiple Pesticides in Basmati Rice

Natasha Logan^1*^, Simon A. Haughey^1^, Lin Liu^1^, D. Thorburn Burns^1^, Brian Quinn^1^, Cuong Cao^1,2^ and Christopher T. Elliott^1^

^1^ASSET Technology Centre, Institute for Global Food Security, School of Biological Sciences, Queen’s University Belfast, 19 Chlorine Gardens, Belfast, BT9 5DL, UK.

^2^Material and Advanced Technologies for Healthcare, Queen’s University Belfast, 18-30 Malone Road, Belfast, BT9 5BN, UK.

* Corresponding author. Email address: N.Logan@qub.ac.uk

**Supplementary Figure 1:** Trend analysis illustrating levels of pesticide residues found in Basmati rice over the Maximum Residue Limits (MRLs) set by the European Union (EU). Data was collected from the RASFF consumer portal and is based on the number of notifications during the period 2011-2020. (a) Percentage of different pesticide residues found in rice over the regulated limits. (b) Percentage of notifications per year for the four most reoccurring residues found in rice.

**Supplementary Table 1:** Current Maximum Residue Limits (MRLs) for pesticide residues in rice by country (mg/kg, ppm).

**Supplementary Table 2:** Characteristic band assignments for acephate, carbendazim, thiamethoxam, tricyclazole and Rhodamine 6G (R6G). The table compares the shift in vibrational bands between Raman and SERS spectra. (ω, ѵ, ѵ_s_, and δ denotes wagging, stretching, symmetric stretching and bending, respectively).

The five major characteristic modes for acephate are 402, 564, 701, 1222 and 1692 cm^-1^ and are assigned to the phosphorous pyramidalization mode, P–S–C mode, P–O–C mode, P=O stretching and the ketone feature, respectively ^1, 2^. The bands between 200 and 400 cm^-1^ are as a result of methyl or oxygen modes, as discussed previously ^3^. In the case of carbendazim, the prominent peaks are at 617, 724, 1006, 1271 and 1474 cm^-1^ and correspond to C–C–C bending, C–H bending in benzene ring, C–O stretching, in-plane C–H bending and C–H/N–H bending, respectively ^4-6^. Whilst, the main vibration bands of thiamethoxam stem from C–N stretching (840 cm^-1^), C–O–C stretching, (984 cm^-1^), C–O stretching (1025 cm^-1^), C=N stretching (1532 cm^-^1) and C=C stretching (1620 cm^-1^) ^7, 8^. Finally, the main characteristic bands of tricyclazole are 418, 593, 974, 1312 and 1371 cm^-1^ (of which 976 cm^-1^ only appears in the SERS spectrum) and are attributed to C–N–C bending, C–S–C bending, C=C stretching and C–N stretching vibration, respectively ^9-11^. Additionally, whilst some peaks are closely related there are some obvious differences between the Raman and SERS spectrum in terms of shifts, bands disappearing or appearing. For example, in the SERS spectrum for tricyclazole various characteristic peaks at 557, 1082, 1225, 1258 and 1414 cm^-1^ appear which are not present in the Raman spectra. However, most bands witness a slight shift in wavenumber (cm^-1^) or sharp increase in SERS intensity after AuNP aggregation.

**Supplementary Figure 2:** Characterisation of gold nanoparticle (AuNP) size using Dynamic Light Scattering (DLS). Z-average (d.nm) was confirmed as (a) 25.4 nm (b) 41.1 nm (c) 56.3 nm and (d) 81.8 nm after synthesis with 0.1%, 0.05%, 0.025% and 0.01% sodium citrate, respectively.

**Supplementary Equation 1:** Determination of Analytical Enhancement Factor (AEF).

Analytical Enhancement Factor (AEF) was used to determine the effectiveness of the developed SERS substrate as a Raman enhancer. AEF can be defined as ^15, 16^ were *I*_RS_ is the Raman signal intensity of an analyte solution at concentration *C*_RS_ under non-SERS conditions and *I*_SERS_ is the SERS intensity measured with a SERS substrate under identical experimental conditions at concentration *C*_SERS_.

**Supplementary Figure 3:** Optimisation of SERS parameters using AuNPs (λ max.= 528 nm). (a) Au substrate concentration. (b) Ratio of Au:pesticide. (c) ‘Hot-spot’ formation reagent. (d) Volume of ‘hot-spot’ reagent. (e) Incubation time. All optimisation was conducted using 100 ppm carbendazim in ethanol:dH_2_O and analysed using handheld-SERS.

The concentration of particles with SPR peak at 528 nm was varied and the results confirmed that the characteristic bands from carbendazim (633, 733, 1006, 1223 and 1265 cm^-1^) were enhanced when the Au concentration was at its highest (OD_528 nm_=3.0) (Fig. SI. 3a). This is attributed to the higher number of particles in solution, therefore more opportunities for aggregation and hot-spot formation. Secondly, the ratio was optimised to promote optimum enhancement. The vibrational carbendazim bands all showed greatest SERS enhancement when the ratio of pesticide:Au was 1:20 thus, allowing sufficient hot-spot formation (Fig. SI. 3b). To achieve maximum SERS enhancement reagents are commonly used to induce aggregation and promote hot-spot formation. These reagents promote hot-spot formation by reducing the inter-particle distance thus, trapping the molecule between adjacent particles. Figure SI. 3c illustrates when 2 M HCl was replaced by 2 M NaCl or 2 M KCl, the SERS intensity was reduced. Therefore, the results confirmed that 2 M HCl (Fig. SI. 3c, blue bars) could initiate hot-spot formation by lowering the pH (most effectively when using a volume of 5 µL (Fig. SI. 3d, blue line)) more effectively than increasing the electrolyte concentration. Finally, the incubation time was studied to allow sufficient time for aggregation and hot-spot formation. Figure SI.3e confirms that a shorter incubation time of 2 min at room temperature provided optimum SERS enhancement, with the intensity of all bands decreasing after this time. However, the bands for CBM are still distinguishable using handheld SERS up to 30 min. The results confirm that the conditions for optimum SERS enhancement are limited by the incubation time, due to the increased level of particle aggregation (or ‘aggregation saturation’) with longer incubations. Thus, for optimum SERS enhancement shorter incubations are preferred. Overall, these experiments could confirm the optimum parameters for handheld-SERS and were applied to all experiments hereafter.

**Supplementary Figure 4:** SERS reference spectra for pesticide residues illustrating their main vibrational bands, analysed using a handheld spectrometer. AuNPs (λ max.= 528 nm) in the presence of HCl (2 M) and 100 ppm (a) acephate, (b) carbendazim, (c) thiamethoxam and (d) tricyclazole.

**Supplementary Table 3:** Limit of detection (LOD), limit of quantification (LOQ), linear function and R^2^ values for the analysis of pesticide residues in solvent conditions and Basmati rice samples, using handheld and benchtop SERS.

**Supplementary Figure 5:** Analysis of pesticide standard solutions (in solvent) using a benchtop Raman microscope. (a, c, e, g) Averaged SERS spectra confirming enhanced SERS intensity with increasing pesticide concentrations. (b, d, f, h) Linear relationship between pesticide concentration and increasing SERS intensity. (a and b) ACE. (c and d) CBM. (e and g) THI. (g and h) TRI. All data was normalized relative to the blank (zero) and the standard deviation (σ) was calculated from triplicate samples (*n=*21).

**Supplementary Figure 6:** Linear relationship between increasing SERS intensity and pesticide concentration extracted from Basmati rice using QuEChERs acetate extraction and analysed using handheld-SERS. Linear range for (a) ACE (b) CBM (c). THI and (d) TRI. All data was normalized relative to the blank (zero) and the standard deviation (σ) was calculated from triplicate samples for each concentration (*n=*18).

**Supplementary Figure 7:** Linear relationship between increasing SERS intensity and pesticide concentration extracted from Basmati rice using QuEChERs acetate extraction and analysed using a benchtop Raman microscope. Linear range (a) ACE (b) CBM (c). THI and (d) TRI. All data was normalized relative to the blank (zero) and the standard deviation (σ) was calculated from triplicate samples for each concentration (*n=*18).

**Supplementary Table 4:** Analytical performance of QuEChERs acetate combined with handheld-SERS used in this work, compared to other methodologies reported in the literature which also focus on detecting pesticide residues in rice.

| **Pesticides tested** | **Matrix** | **Technique** | **Linear range (ppb)** | **Sensitivity (ppb)** | **Recovery (%)** | **RSD (%)** | **Portability (Y/N)** | **Reference** |
| --- | --- | --- | --- | --- | --- | --- | --- | --- |
| acephate, carbendazim, thiamethoxam, tricyclazole | Basmati rice | Handheld-SERS | 1 - 10000 | 0.61 - 825 | 83.4 – 115.0 | 3.6 – 23.8 | Y | This work |
| 42 pesticides | Polished rice | LC-MS/MS | 50 – 500 | 0.1 – 10 | 70 - 120 | < 20 | N | [17] |
| 124 pesticides | Rice | GC-MS/MS | 10 - 200 | 0.1 – 7.9 | 70 – 122.7 | < 20 | N | [18] |
| acephate | Rice | D-SERS | 500 - 100200 | 500 | NA | 4.0 | Y | [19] |
| carbendazim | NA | SERS | 190 - 9600 | 9600 | NA | NA | N | [20] |
| tricyclazole | Paddy rice | SERS | 50 - 700 | 2.0 | 88.3 – 104.8 | 3.63 – 4.64 | Y | [21] |
| chlorpyriphos | Rice | SERS | 500 - 50000 | 506 | 96.59 – 104.69 | 2.64 – 4.47 | Y | [22] |

**Supplementary Table 5:** Recovery (mg/kg, ppm), relative standard deviation (%) and relative error (%) of pesticide residues extracted from Basmati rice using QuEChERs acetate analysed using handheld and benchtop SERS.

* **RE** - relative error (RE_precision_) between the two SERS techniques

**Supplementary References**

1. Clauson, S. L., Sylvia, J. M., Arcury, T. A., Summers, P. & Spencer, K. M. Detection of Pesticides and Metabolites Using Surface-Enhanced Raman Spectroscopy (SERS): Acephate. *Appl. Spectrosc* **69**, 785-793 (2015).

2. Tanner, P. A. & Leung, K.-H. Spectral Interpretation and Qualitative Analysis of Organophosphorus Pesticides Using FT-Raman and FT-Infrared Spectroscopy. *Appl. Spectrosc* **50**, 565-571 (1996).

3. Zhang, Y., et al. A first principle study of terahertz (THz) spectra of acephate. *Chem. Phys. Lett* **452**, 59-66 (2008).

4. Strickland, A. D & Batt, C. A. Detection of Carbendazim by Surface-Enhanced Raman Scattering Using Cyclodextrin Inclusion Complexes on Gold Nanorods. *Anal. Chem* **81**, 2895-2903 (2009).

5. Chen, X., et al. Detection and quantification of carbendazim in Oolong tea by surface-enhanced Raman spectroscopy and gold nanoparticle substrates. *Food Chem* **293**, 271-277 (2019).

6. Furini, L. N., et al. Detection and quantitative analysis of carbendazim herbicide on Ag nanoparticles via surface-enhanced Raman scattering. *J. Raman Spectrosc* **46**, 1095-1101 (2015).

7. Gao, Y., Xu, M.-L. & Xiong, J. Raman and SERS spectra of thiamethoxam and the Ag3–thiamethoxam complex: an experimental and theoretical investigation. *J. Environ. Sci. Health B* **54**, 665-675 (2019).

8. Atanasov, P. A., et al. SERS analyses of thiamethoxam assisted by Ag films and nanostructures produced by laser techniques. *J. Raman Spectrosc* **49**, 397-403 (2018).

9. Chen, K., Shen, Z., Luo, J., Wang, X. & Sun, R. Quaternized chitosan/silver nanoparticles composite as a SERS substrate for detecting tricyclazole and Sudan I*. Appl. Surf. Sci* **351**, 466-473 (2015).

10. Li, Q.-Q., et al. Rapid and sensitive detection of pesticides by surface-enhanced Raman spectroscopy technique based on glycidyl methacrylate–ethylene dimethacrylate (GMA–EDMA) porous material. *Chin. Chem. Lett* **24**, 332-334 (2013).

11. Tang, H. R., et al. Surface enhanced Raman spectroscopy signals of mixed pesticides and their identification. *Chin. Chem. Lett* **22**, 1477-1480 (2011).

12. Chen, W., et al. Rapid and sensitive detection of pesticide residues using dynamic surface-enhanced Raman spectroscopy. *J. Raman Spectrosc* **51**(4), 611-618 (2020).

13. Lu, Y., Liu, G. L. & Lee, L. P. High-Density Silver Nanoparticle Film with Temperature-Controllable Interparticle Spacing for a Tunable Surface Enhanced Raman Scattering Substrate. *Nano Lett* **5,** 1, 5-9 (2005).

14. Jensen, L. & Schatz, G. C. Resonance Raman Scattering of Rhodamine 6G as Calculated Using Time-Dependent Density Functional Theory. *J. Phys. Chem. A* **110**, 18, 5973-5977 (2006).

15. Le Ru, E. C., Blackie, E., Meyer, M. & Etchegoin, P. G. Surface Enhanced Raman Scattering Enhancement Factors:  A Comprehensive Study. *J. Phys. Chem. C* **111**, 13794-13803 (2007).

16. Akanny, E., et al. Development of uncoated near-spherical gold nanoparticles for the label-free quantification of Lactobacillus rhamnosus GG by surface-enhanced Raman spectroscopy. *Anal. Bioanal. Chem* **411**, 5563-5576 (2019).

17. Pareja, L., Cesio, V., Heinzen, H. & Fernández-Alba, A. R. Evaluation of various QuEChERS based methods for the analysis of herbicides and other commonly used pesticides in polished rice by LC–MS/MS. *Talanta* **83**, 1613-1622 (2011).

18. Hou, X., Han, M., Dai, X., Yang, X. & Yi, S. A multi-residue method for the determination of 124 pesticides in rice by modified QuEChERS extraction and gas chromatography–tandem mass spectrometry. *Food Chem*. **138**, 1198-1205 (2013).

19. Weng, S., et al. Dynamic surface-enhanced Raman spectroscopy for the detection of acephate residue in rice by using gold nanorods modified with cysteamine and multivariant methods. *Food Chem*. **310**, 125855 (2020).

20. Strickland, A. D. & Batt, C. A. Detection of carbendazim by surface-enhanced Raman scattering using cyclodextrin inclusion complexes on gold nanorods. *Anal. Chem*. **81**, 2895-2903 (2009).

21. Tang, H., et al. Determination of tricyclazole content in paddy rice by surface enhanced Raman spectroscopy. *J Food Sci*. **77**, 105-109 (2012).

22. Huang, S., Hu, J., Guo, P., Liu, M. & Wu, R. Rapid detection of chlorpyriphos residue in rice by surface-enhanced Raman scattering. *Anal. Methods* **7**, 4334-4339 (2015).
